# Bronchiectasis Assessment in Primary Ciliary Dyskinesia: A Non-Invasive Approach Using Forced Oscillation Technique

**DOI:** 10.3390/diagnostics13132287

**Published:** 2023-07-06

**Authors:** Wilfredo De Jesús-Rojas, Luis Reyes-Peña, José Muñiz-Hernández, Patricia Quiles Ruiz de Porras, Jesús Meléndez-Montañez, Marcos J. Ramos-Benitez, Ricardo A. Mosquera

**Affiliations:** 1 Department of Pediatrics and Basic Science, Ponce Health Sciences University, Ponce, PR 00716, USA; jomuniz@psm.edu (J.M.-H.); patriciaqr@sanjuanbaustista.edu (P.Q.R.d.P.); mjramos@psm.edu (M.J.R.-B.); 2 San Juan Bautista School of Medicine, Caguas, PR 00725, USA; luisrp@sanjuanbautista.edu; 3 Department of Pediatrics, McGovern Medical School, University of Texas Health Science Center at Houston, Houston, TX 77030, USA; ricardo.a.mosquera@uth.tmc.edu

**Keywords:** forced oscillation technique, bronchiectasis, primary ciliary dyskinesia, respiratory impedance, respiratory mechanics, preliminary research

## Abstract

Primary ciliary dyskinesia (PCD) is an autosomal recessive disorder that results from the dysfunction of motile cilia, which can cause chronic upper and lower respiratory infections leading to bronchiectasis. However, there is a need for additional tools to monitor the progression of bronchiectasis in PCD. The forced oscillation technique (FOT) is an effort-independent lung function test that can be used to evaluate respiratory mechanics. In this retrospective study, we aimed to describe the radiographic findings associated with respiratory impedance (resistance (Rrs) and reactance (Xrs)) measured by FOT in six adult PCD patients and one pediatric with the (*RSPH4A* (c.921+3_921+6delAAGT (intronic)) founder mutation. We compared the radiographic findings on a high-resolution chest computed tomography (CT) scan with the FOT results. Our findings suggest that respiratory impedance measured by FOT may be a valuable tool for detecting and monitoring the progression of bronchiectasis in PCD patients with the (*RSPH4A* (c.921+3_921+6delAAGT (intronic)) founder mutation. However, further research is necessary to validate these results and determine the sensitivity and specificity of bronchiectasis monitoring in PCD patients with other genetic mutations.

## 1. Introduction

Primary ciliary dyskinesia (PCD) is an autosomal recessive heterogenous disorder caused by the dysfunction of motile cilia, which are responsible for clearing mucus and foreign material from the airways [1,2]. The motile cilia play an essential role in clearing mucus and other debris from the lungs, allowing for proper respiratory function [3]. In PCD, the cilia are either absent or do not work correctly, resulting in recurrent infections of the upper and lower respiratory tracts [4]. The hallmark symptom of PCD is a chronic wet cough, which is often accompanied by excess mucus production [5]. Over time, this chronic inflammation and mucus accumulation can lead to irreversible lung damage and bronchiectasis, a condition in which the airways become permanently widened and scarred [6]. Bronchiectasis in PCD is an irreversible medical condition characterized by permanent enlargement and destruction of bronchi and bronchioles [7]. Respiratory symptoms secondary to bronchiectasis in PCD could include shortness of breath, wheezing, and chest pain and can lead to respiratory failure in severe cases [8]. Due to delayed diagnosis and treatment, PCD patients often experience worsened prognoses [9]. Therefore, there is a need for more tools that can aid in the early detection and management improvement of PCD comorbidities [3]. Recent studies have found that forced oscillation technique (FOT) results can be a viable indicator of bronchiectasis severity in patients with non-cystic fibrosis (CF) and non-PCD bronchiectasis [10].

FOT is an effort-independent lung function test that evaluates respiratory mechanics, specifically airway resistance (Rrs) and reactance (Xrs) and has shown promise in the diagnosis and treatment of respiratory diseases, such as asthma [11,12]. With its noninvasive and effort-independent method, FOT is of great interest for use in pediatric and geriatric populations who may be unable to perform other commonly used lung function tests, such as spirometry [13,14]. Spirometry measures the volume and speed of air that a person can inhale and exhale, while FOT measures the resistance and reactance of airflow through the lungs. Spirometry provides values for forced vital capacity (FVC), forced expiratory volume in one second (FEV1), and the ratio of FEV1 to FVC, commonly used to diagnose and monitor lung diseases, such as PCD. In FOT, the Rrs and Xrs are two important parameters that provide information on the respiratory mechanics of an individual. Rrs is a measure of the opposition to airflow in the respiratory system. It reflects the resistance of the airways, lung tissue, and chest wall and is influenced by various factors, such as airway caliber, airway wall thickness, and lung tissue elasticity. Xrs, on the other hand, represents the capacity of the respiratory system to store and release energy during the breathing cycle. It reflects the elastic properties of the lung tissue and the airways and can provide information on the presence of obstruction or restriction in the respiratory system. In FOT, both Rrs and Xrs are measured across a range of frequencies, and their values can provide insights into the mechanical properties of the respiratory system, including the presence of inflammation, airway narrowing, and air trapping [15], which can be helpful in assessing the severity and progression of lung diseases.

Despite the promising results of FOT in assessing respiratory function in various conditions, including asthma and COPD, its application in PCD has not been studied extensively. The need for research is especially relevant considering the high prevalence of bronchiectasis in PCD patients and the lack of effective non-invasive methods for assessing the severity of lung damage in these patients. Therefore, in this study, we aimed to investigate the potential utility of FOT as a non-invasive tool for assessing the severity of bronchiectasis in patients with PCD at different stages of lung damage. By comparing FOT results with radiographic imaging findings, we aimed to fill the gap in the literature on the use of FOT in patients with PCD and investigate its potential as an early detection and clinical management tool for PCD comorbidities. However, more research is needed to explore the use of FOT with other respiratory conditions and to establish the correlation between FOT values and disease progression in PCD patients.

## 2. Materials and Methods

A retrospective chart review was conducted of one pediatric and six adult patients with PCD homozygous for the (*RSPH4A* (c.921+3_921+6delAAGT (intronic)) founder mutation [16], who were seen at the Puerto Rico PCD center [17]. We assessed FVC, FEV1, FEV1/FVC, forced expiratory flow at 25% and 75% of the pulmonary volume (FEF 25–75%), and FEF max following the American Thoracic Society (ATS) guidelines [18]. Airflow limitation in this study was assessed using Z-score values with a threshold of −1.64 as the lower limit of normal. The reference equation used for this assessment was the Global Lung Function Initiative-2012 (GLI) equation [19]. FOT was performed using the Resmon™ Pro V3 device (RESTECH Srl, Milano, Italy) to measure resistance [cmH_2_O/(s/L)] and reactance [cmH_2_O/(s/L)]. The FOT involved 10 acceptable breaths at 8 Hz for pediatric patients and in a multi-frequency mode of 5-11-19 Hz for adult patients as established by the European Respiratory Society (ERS) [15]. After establishing baseline values for each patient, FOT was repeated after bronchodilation therapy with an albuterol sulfate inhalation solution of 2.5 mg/3 mL single dose. A significant response was defined as a 40% change in FOT from baseline following bronchodilation. In addition to reviewing respiratory impedance measured by FOT, we retrospectively analyzed high-resolution chest CT (HRCT) scans to evaluate the radiographic findings associated with bronchiectasis in our PCD patients.

## 3. Results

The study included a population of seven patients with PCD, who had the (*RSPH4A* (c.921+3_921+6delAAGT (intronic)) founder mutation. The median age of the patients was 26 years, and the majority of them were female (85.7%) and of Hispanic Puerto Rican ethnicity. The study found that most patients (71.4%) had a history of asthma, while all patients experienced year-round wet cough and daily nasal congestion as PCD-related symptoms. Neonatal respiratory distress was reported in 42.8% of patients. Bronchiectasis, a common complication of PCD, was observed in all patients (100%), and 14.2% of patients had a history of allergic bronchopulmonary aspergillosis (ABPA). Moreover, 85.7% of patients had a history of *Pseudomonas* spp. infection. Table 1 provides a summary of demographic data. These findings highlight the clinical characteristics of the PCD patient population in the study and provide insight into the prevalence of common complications associated with the disease.

Table 2 presents the baseline spirometry results of seven patients with PCD with the (*RSPH4A* (c.921+3_921+6delAAGT (intronic)) founder mutation. The spirometry results showed abnormal values for most of the patients, with two cases exhibiting airflow obstruction and four cases suggesting a possible restrictive airflow pattern. FVC values ranged from 37% to 94% of predicted values, with Case G having a normal FVC of 94%. FEV1 values ranged from 33% to 85% of predicted values, with Cases E and F exhibiting an obstructive pattern. The FEV1/FVC ratio ranged from 64% to 107%, with most patients having values within the normal range. The FEF 25–75% values ranged from 17% to 62% of predicted values, with most patients exhibiting values below the normal range. The FEF max values ranged from 34% to 87% of predicted values, with most patients exhibiting values below the normal range.

**Figure 1:**
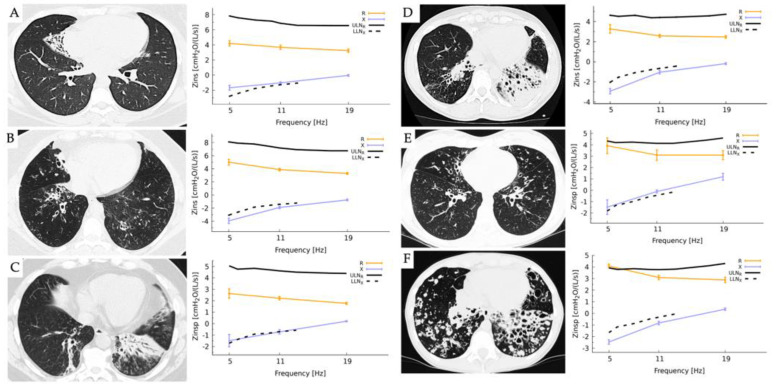
Results of measurements obtained by the forced oscillatory technique using the Resmon™ Pro V3 device in six adult patients with primary ciliary dyskinesia (PCD) who were homozygous for the (*RSPH4A* (c.921+3_921+6delAAGT (intronic)) founder mutation. The FOT was completed with 10 acceptable breaths at a multi-frequency mode of 5-11-19 Hz to measure resistance (Rrs) [cmH_2_O/(s/L)] and reactance (Xrs) [cmH_2_O/(s/L)]. High-resolution chest CT (HRCT) scans are presented as a representative example in each case. In the figure, the solid bar represents the upper limit of normal for resistance (ULN_R_), while the intermittent dotted line represents the lower limit of normal for reactance (LLN_x_).

Figure 1 presents six cases of bronchiectasis with varying characteristics and the respective FOT values. Case A: a 24-year-old female with a right lower wedge area of lobar bronchiectasis with both normal Rrs and reactance Xrs (Rrs < ULN_R_ and Xrs > LLNx). Case B: a 54-year-old female with bibasilar bronchiectasis more prominent at the right lower lobes. Case C: a 51-year-old male with bibasilar atelectasis and bronchiectasis more prominent on the left lower lobe. Case D: a 59-year-old female with bilateral basal bronchiectasis more pronounced at the LLL. Cases B, C, and D showed normal Rrs, but Xrs was abnormal (Rrs < ULN_R_ and Xrs < LLNx), suggesting a peripheral disease, disomogeneity of ventilation, or possible restriction. Case E is a 17-year-old female with central and bibasilar bronchiectasis; FOT results showed borderline Rrs and Xrs. Case F: a 23-year-old female with severe cylindrical bronchiectasis bilaterally affecting lower lobes, right middle, and lingula. In this case, both Rrs and Xrs were outside the normal range (Rrs > ULN_R_ and Xrs < LLNx), consistent with obstructive lung disease.

**Figure 2:**
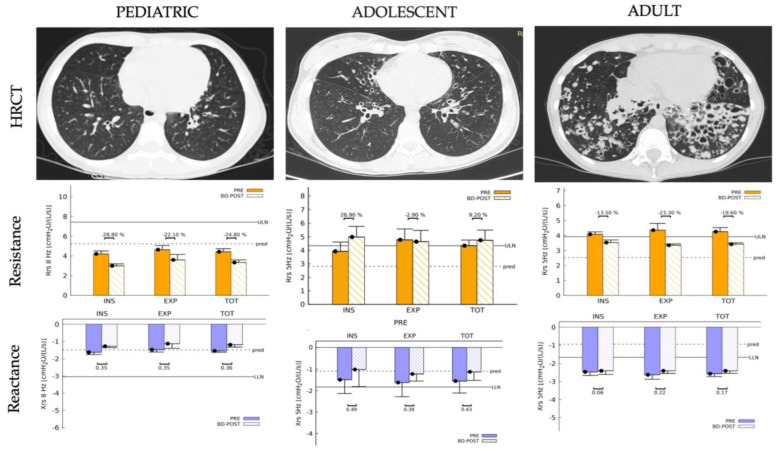
Comparison of high-resolution chest CT (HRCT) scans findings and lung resistance (Rrs) and reactance (Xrs) measured by forced oscillation technique (FOT) among a pediatric (Case G), adolescent (Case E), and adult patient (Case F) with primary ciliary dyskinesia (PCD) due to the (*RSPH4A* (c.921+3_921+6delAAGT (intronic)) founder mutation. In the figure, the solid bars indicate the upper limit of normal for resistance (ULN_R_) and the lower limit of normal for reactance (LLN_x_). The intermittent dotted line represents the predicted values in each case.

Figure 2 presents three cases, a 14-year-old female pediatric patient (Case G) HRCT scan with minimal bibasilar bronchiectasis and normal Rrs and Xrs, an adolescent patient (Case E) with HRCT showing moderate bronchiectasis with increased Rrs and normal Xrs, and an adult patient (Case F) HRCT scan that shows severe bibasilar cylindrical bronchiectasis with increased Rrs and Xrs. No significant changes in Rrs or Xrs were observed post-bronchodilator in the pediatric or adult cases.

## 4. Discussion

Through the comparison of data visualization from FOT and HRCT results, we found that FOT measurements have the potential to aid in the monitoring of the development and progression of bronchiectasis disease in PCD patients. As PCD is a chronic condition, ongoing monitoring of respiratory function is essential to identify bronchiectasis progression, assess treatment efficacy, and optimize patient management. Traditionally, spirometry has been the standard method for assessing lung function in patients with respiratory diseases, including PCD. However, spirometry alone may not provide a comprehensive assessment of respiratory function in PCD as it primarily measures airflow limitation and may not capture other important aspects of respiratory function, such as airway distensibility and reactance of the lungs. FOT is a non-invasive method that provides additional insights into respiratory function by measuring impedance, which consists of resistance and reactance. FOT can assess airway distensibility, peripheral airway function, and central and peripheral obstruction in PCD, which may not be captured by spirometry alone. Table 3 provides a comparison between spirometry and FOT techniques.

Previous studies have demonstrated the efficacy of FOT in evaluating respiratory function in various respiratory diseases, such as asthma [20], chronic obstructive pulmonary disease (COPD) [21], and cystic fibrosis [22]. The non-invasive nature of FOT allows for repeated measurements and may provide a more comprehensive assessment of respiratory mechanics compared to other pulmonary function tests. However, there have been no studies evaluating the use of FOT in patients with PCD, making this study the first to investigate the potential of FOT in this patient population. As such, the findings of this study may have important implications for the clinical management and monitoring of PCD patients, demonstrating that further research in this area is warranted.

The baseline spirometry data, as presented in Table 2, showed abnormal values for most of the reported cases, with two cases exhibiting airflow obstruction and four cases suggesting a possible restrictive airflow pattern. These findings are consistent with previous studies showing lower FEV1 in adults with PCD compared to children [23]. Additionally, patients with lower FEV1 demonstrated abnormal values for airway resistance and reactance, particularly in cases with moderate to severe bronchiectasis lung disease, as observed in Cases E and F in Figure 1, which aligns with findings in individuals with COPD showing an inverse association between respiratory impedance and spirometry parameters [24].

Previous studies on patients with non-PCD bronchiectasis have reported a strong correlation between FOT parameters and the severity of bronchiectasis disease [25]. Another study highlighted that total breath reactance at 5 Hz (X_5_) could effectively differentiate between healthy subjects and patients with obstructive pulmonary diseases [21]. Additionally, patients with COPD exhibited an increased change in R_5_-R_20_ results, which could aid in distinguishing between abnormal FOT results caused by COPD and bronchiectasis [21]. Consistent with these findings, our study also observed abnormal X_5_ FOT results in patients with moderate to severe PCD bronchiectasis, aligning with the observations made by Dos Santos et al. [26].

According to the findings of Tan C. et al., bronchiectasis severity scores exhibited positive correlations with various FOT parameters, including R_5_, R_5_-R_20_, resonance frequency, airway impedance at 5 Hz, peripheral resistance, and negative correlation with X_5_ [25]. In our study, we utilized the multi-frequency mode of FOT to measure pulmonary resistance and reactance at frequencies 5-11-19 Hz. We observed borderline normal to abnormal reactance values at frequencies 5-11-19 Hz in patient cases B, C, D, and F, as presented in Figure 1. These results suggest that abnormal values for reactance (Xrs) at frequencies 5-11-19 Hz may serve as a possible indicator for evaluating the development of bronchiectasis in patients with PCD. We also observed an increasing trend in the values of R_5_ in cases with moderate to severe bronchiectasis disease (Cases E and F, respectively), as seen in Figure 1, suggesting that R_5_ may be a potential measurement in tracking the progression of lung damage.

Additional studies are needed to fully understand the progression of lung disease in PCD as some patients may experience end-stage lung disease in their middle-aged years, emphasizing the importance of early detection and diagnosis [27]. Currently, there are limited tools for studying and evaluating the progression of lung disease in patients with PCD beyond spirometry measurements and imaging studies requiring radiation, such as CT scans. FOT may present as a feasible non-invasive tool that provides additional information on a patient’s respiratory system function and mechanics [28,29]. FOT does not involve radiation and requires little or no respiratory effort from the patient, making it a viable alternative for pediatric and elderly patients and those with advanced respiratory impairment [30]. Further research on the use of FOT in bronchiectasis assessment for patients with PCD is essential in improving the clinical management of the disease. Longitudinal studies can provide insights into the ability of FOT to monitor lung function decline over time, which can help identify patients at high risk of disease progression. The early identification of patients at risk of lung function decline can help healthcare providers implement early interventions and improve patient outcomes. By tracking changes in lung function over time, clinicians can determine the effectiveness of interventions and adjust individualized treatment plans.

The use of FOT in clinical trials may be important in determining the efficacy of various interventions in improving respiratory function in PCD patients. One example of a clinical trial that could use FOT as an outcome measure is the study of airway clearance techniques in PCD patients. FOT can be used as a variable to assess the effectiveness of different airway clearance techniques in improving respiratory function in PCD patients. Another clinical trial that can use FOT as an outcome measure is the study of medications, including antibiotics and anti-inflammatory agents, in improving lung function in PCD patients pre-and post-treatment. FOT can be used to monitor changes in lung mechanics over time and to evaluate the effectiveness of different medications in managing PCD.

Other studies, including correlation studies, can investigate the relationship between FOT measurements and other clinical outcomes, such as quality of life, exacerbation rates, and hospitalizations. These studies can provide valuable information on the utility of FOT in monitoring disease progression and treatment response in PCD patients. Finally, cost-effectiveness studies can evaluate the economic impact of using FOT for bronchiectasis assessment in PCD patients. This can help establish the value of FOT in the clinical management of PCD and aid in the development of reimbursement policies. By understanding the economic impact of using FOT, healthcare providers can make informed decisions on the appropriate use of this technology in PCD patient care.

However, it should be noted that FOT has limitations, such as the reliance on reference values established in specific populations, which may limit generalizability to other groups. [12]. Further research is needed to establish appropriate reference values for FOT in diverse populations, including those with chronic respiratory diseases, such as PCD. To understand more about the value of FOT in PCD, it is important to establish normative data for FOT in PCD patients. It is crucial in interpreting FOT measurements to conduct studies on a larger scale to create accurate predictive values in the PCD population and across different genetic mutations. This can help improve the clinical management of PCD patients and aid in the early detection of bronchiectasis. By establishing normative data, healthcare providers can better understand the range of FOT values in PCD patients, allowing for more accurate diagnoses and monitoring of disease progression in the future.

Additionally, the acquisition of normative data will help in the development of future clinical guidelines and protocols for the use of FOT in PCD patients. Additionally, our study has limitations, including the rarity of PCD, the focus on a specific PCD genetic variant (*RSPH4A* (c.921+3_921+6delAAGT (intronic)), and the need for more extensive prospective studies that encompass diverse PCD mutations to improve generalizability.

## 5. Conclusions

The retrospective study conducted on seven PCD patients with the *RSPH4A* (c.921+3_921+6delAAGT (intronic)) founder mutation provides valuable insight into the potential of FOT as a tool to monitor bronchiectasis development and progression in PCD patients. The results suggest that FOT can provide additional information on respiratory mechanics that could be used to complement standard pulmonary evaluations. This is particularly important as bronchiectasis is a progressive disease, and early detection and intervention can significantly improve the clinical outcome for patients.

While the study findings are promising, further research is necessary to establish a clear correlation between FOT values and lung disease progression in PCD patients. Future studies could investigate the ability of FOT to track the progression of bronchiectasis over time and identify patients at high risk of lung function decline. Additionally, comparison studies between FOT and other lung function tests, such as spirometry and CT scans, can provide a better understanding of the additional information provided by FOT measurements. Furthermore, longitudinal studies and clinical trials could help evaluate the effectiveness of various interventions, such as airway clearance techniques and medications, in improving respiratory function in PCD patients, with FOT used as an outcome measure.

Overall, the retrospective study highlights the potential of FOT as a non-invasive tool to monitor the progression of lung disease in PCD patients. The study results suggest that FOT could complement standard pulmonary evaluations and provide additional information on respiratory mechanics. However, further research is necessary to establish the efficacy of FOT in tracking the progression of bronchiectasis and its ability to identify patients at high risk of lung function decline. If successful, FOT could significantly improve the clinical management of PCD and aid in the early detection and intervention of bronchiectasis.

## Figures and Tables

**Table 1:** Demographic data and clinical characteristics of the seven PCD patients with the (*RSPH4A* (c.921+3_921+6delAAGT (intronic)) founder mutation included in this study.

Characteristics	Mean (% of *n* = 7)
Age, median ± IQR	26 ± 24.5
Gender	
Female	6 (85.7)
Male	1 (14.3)
Ethnicity	
Hispanics, Puerto Ricans	7 (100)
(*RSPH4A* (c.921+3_921+6delAAGT (intronic))	7 (100)
Asthma	5 (71.4)
PCD-related symptoms:	-
Year-round wet cough	7 (100)
Year-round daily nasal congestion	7 (100)
Neonatal respiratory distress	3 (42.8)
Bronchiectasis	7 (100)
ABPA ^1^	1 (14.2)
History of *Pseudomonas* spp. infection	6 (85.7)

^1^ ABPA: allergic bronchopulmonary aspergillosis. All values are represented in percentages except for age, represented in the median.

**Table 2:** Baseline spirometry results of patients with PCD due to the (*RSPH4A* (c.921+3_921+6delAAGT (intronic)) founder mutation.

Cases	Age (Years)	FVC	FEV1	FEV1/FVC	FEF 25–75	FEF Max	Airflow
A	24	72 *	72 *	99	73	76	Restrictive
B	54	64 *	51 *	80	25 *	55 *	Restrictive
C	51	47 *	43 *	92	35 *	39 *	Restrictive
E	59	65 *	40 *	64 *	19 *	34 *	Obstructive
D	17	37 *	39 *	107	50 *	69 *	Restrictive
F	23	44 *	33 *	75	17 *	42 *	Obstructive
G	14	94	85	91	62 *	87	Normal

Forced vital capacity (FVC), forced expired volume in 1 s (FEV1), FEV1 to FVC ratio (FEV1/FVC), forced expiratory flow at 25 and 75% of the pulmonary volume (FEF 25–75%), and maximum forced expiratory flow (FEF Max) taken following the American Thoracic Society (ATS) guidelines. All values are represented in percentages predicted for age. (*) Values were considered abnormal if less than 80% of predicted.

**Table 3:** Comparison of spirometry and FOT technique.

	Spirometry	Forced Oscillation Technique (FOT)
Measures	Forced vital capacity (FVC), forced expiratory volume in 1 s (FEV1), FEV1/FVC ratio, maximum forced expiratory flow (FEF Max).	Resistance (Rrs) and reactance (Xrs) at multiple frequencies.
Technique	Measures airflow during forced exhalation.	Measures resistance and reactance of airways during quiet breathing. Does not require maximal effort from the patient. Can be used to measure airway function in young children and patients with severe lung disease.
Limitations	May not detect early airway obstruction. Limited usefulness in patients with severe lung disease.	Limited reference values in certain populations. May not be as widely available as spirometry. Limited ability to measure gas exchange and lung volumes.

## Data Availability

All data are available upon request through the corresponding author.
